# Dentinal Anesthesia

**Published:** 1904-01-15

**Authors:** 

**Affiliations:** Bonn


### DENTINAL ANESTHESIA.

BY DR. THIESING, BONN.

Dr. Thiesing recommends the use of ammonia solutions for dentinal anesthesia. His experiments have demonstrated that these solutions in a majority of cases bring about anesthesia of the dentin. The objectionable properties of solutions of ammonia salts, which preclude their being used hypodermically, disappear completely when used to obtund sensitive dentin. The anesthesia of the teeth lasts from a few hours to a few days, according to the strength of the solution employed and to the length of time during which it is allowed to remain in the tooth. These solutions do not discolor the teeth and do not have any irritating action on the pulp. The objectionable and penetrating odor can be attenuated by the addition of a solution of etheral oil in alcohol. Ammonium bromid, in ten percent solutions, produces a very feeble degree of anesthesia. Ammonium chlorid, in twenty percent solutions, after ten minutes' contact, produces slight anesthesia and no pain. Ammonium phosphid produces slight pain and complete anesthesia after five to ten minutes. Ammonium acetate produces complete anesthesia after five minutes, but at the same time it causes severe pain, which, however, lasts only a few seconds. Ammonium carbonate, in solutions of five to twenty percent, causes no pain and complete anesthesia after five to ten minutes. This is the solution recommended by the author. Caustic ammonia in solutions of one-fourth of one percent to which is added eight-tenths of one percent of sodium chlorid, does not anesthetize dentin after five minutes, but after ten minutes brings about a sufficient degree of insensitivity. The application of this solution is not painful, and solutions of ten to twenty percent always produce dentinal insensitivity after five to ten minutes. Solutions of thirty to fifty percent produce good results; these are, however, accompanied by pain, which can be avoided by the addition of concentrated carbolic acid.

